# Home-prepared Hamburger and Sporadic Hemolytic Uremic Syndrome, Argentina

**DOI:** 10.3201/eid0909.020563

**Published:** 2003-09

**Authors:** Marta Rivas, María Gracia Caletti, Isabel Chinen, Stella Maris Refi, Carlos Daniel Roldán, Germán Chillemi, Graciela Fiorilli, Alicia Bertolotti, Lorena Aguerre, Sergio Sosa Estani

**Affiliations:** * Instituto Nacional de Enfermedades Infecciosas-ANLIS “Dr. Carlos G. Malbrán,” Buenos Aires, Argentina.; † Hospital Nacional de Pediatría “Prof. Dr. Juan Garrahan,” Buenos Aires, Argentina; ‡ Laboratorio Central de Salud Pública, La Plata, Argentina; § Centro Nacional de Diagnóstico e Investigación en Endemoepidemias-ANLIS “Dr. Carlos G. Malbrán,” Buenos Aires, Argentina

**Keywords:** HUS, *Escherichia coli* O157, ground beef, Argentina

**To the Editor:** Argentina has the highest incidence of hemolytic uremic syndrome (HUS) in the world, and 10.4 cases per 100,000 children <5 years of age were reported in 2001. HUS is the leading cause of acute renal failure in children (*1*); in 20% to 35% serious chronic renal failure develops, ranging from mild to serious, and HUS is the second leading cause of chronic renal failure (*2*,*3*) in Argentina. Recently, evidence of Shiga toxin–producing *Escherichia coli* (STEC) infection was found in 59% of Argentine HUS cases; O157:H7 was the predominant serotype isolated (*4*). Although outbreaks of *E. coli* O157:H7 have been linked to eating contaminated ground beef (*5*), the organism is rarely isolated from the implicated meat, and the sources of infection for sporadic cases have rarely been identified. We report a sporadic HUS case linked to the consumption of home-prepared hamburger contaminated with *E. coli* O157.

A 2-year-old girl was brought to the emergency room of the Hospital Nacional de Pediatría “Prof. Dr. Juan Garrahan” in Buenos Aires on April 26, 2002, with a 1-day history of bloody diarrhea. Results of a physical examination were normal, and a stool culture was requested. The patient was sent home with dietary and general instructions. As watery diarrhea persisted with vomiting and fever, the girl was brought in again 3 days later. At that time, she exhibited moderate dehydration, pallor, drowsiness, and a generalized seizure of 10 to 15 min duration, tachycardia, tender and tense abdominal wall, and a history of oligoanuria for the last 48 h. Blood pressure was 128/67 mm Hg. The child was hospitalized with a presumptive diagnosis of HUS and anuric renal failure.

Initial laboratory findings included the following: hematocrit, 26%; hemoglobin level, 8.8 g/dL; leukocyte count, 34,800/mm^3^; segmented neutrophil count, 29,928/mm^3^; platelet count, 91,000/mm^3^; serum glucose, 160 mg/dL; blood urea nitrogen (BUN), 268 mg/dL; serum creatinine, 6.3 mg/dL; albumin, 1.7 g/dL; uric acid**,** 14.8 mg/dL; calcium, 6.9 mg/dL; phosphorus, 6.7 mg/dL; magnesium, 2.0 mg/dL; sodium, 113 mEq/L; potassium, 7.6 mEq/L; pH 7.28; bicarbonate, 10 mmol/L; base excess, –14.9 mmol/L. Chest x-ray findings were normal with a cardiothoracic index of 0.5; results of an abdominal sonogram were normal. A sonogram of the renal system also showed that the kidneys were of normal shape and size and had increased echogenicity. Results of a brain scan showed nonspecific brain atrophy**.**

The clinical findings and the laboratory features of microangiopathic hemolytic anemia, thrombocytopenia, and acute renal failure were consistent with the diagnosis of HUS. The patient remained anuric for 17 days, required 17 peritoneal dialysis procedures, and six infusions of packed red blood cells. One month after the acute period, she had elevated BUN and serum creatinine levels and massive proteinuria.

The rectal swab sample collected on April 26 was routinely cultured for *E. coli*, *Salmonella*, *Shigella*, *Yersinia*, *Aeromonas*, *Plesiomonas*, *Vibrio,* and *Campylobacter* species. Sorbitol nonfermenting colonies were recovered on sorbitol-MacConkey (SMAC) agar (Difco Laboratories, Detroit, MI) and SMAC supplemented with cefixime (50 ng/mL) and potassium tellurite (25 mg/mL) (CT-SMAC). The bacterial confluent growth zones of both SMAC and CT-SMAC were positive for *stx*2 and *rfb*O157 genes by multiplex polymerase chain reaction (PCR) using the primers described by Pollard et al. (*6*) and Paton et al. (*7*), respectively. The *E. coli* O157 isolates were identified by standard biochemical methods and serologic tests by using specific O157 antiserum (INPB-ANLIS “Dr. Carlos G. Malbrán”) and sent to the Servicio Fisiopatogenia as National Reference Laboratory (NRL) for further characterization.

As part of the case-control study conducted in the pediatric hospital to identify the risk factors associated with the STEC infection, parents of the 2-year-old girl were interviewed with a standardized questionnaire 8 days after onset of symptoms. Information was collected about her clinical illness, potential exposures in the 7 days before the onset of diarrhea, and demographic issues. Her parents reported that on April 25 the girl had eaten a home-prepared hamburger, made from ground beef purchased from a local market. No other family members reported diarrhea.

Three days after the interview, on May 6, a formal complaint was presented by the mother at the Division of Public Health of Lanús, in the southern area of Buenos Aires, where the family lives. The frozen leftover ground beef from the same package used to make the hamburgers was provided by the child’s family and processed at the Laboratorio Central de Salud Pública.

A 65-g portion of the ground beef was incubated overnight at 42°C in 585 mL of modified *E. coli* medium broth containing novobiocin (final concentration, 20 μg/mL). The sample was positive using the E. coli O157 Visual Immunoassay (Tecra Internacional Pty. Ltd., French Forest NSW, Australia) (*8*). Immunomagnetic separation was performed with 1 mL of the culture, according to the instructions of the manufacturer (Dynal, Inc., Oslo, Norway). The concentrate sample was plated onto CT-SMAC and O157:H7 ID medium (bioMérieux, Marcy l’Etoile, France). Up to 20 sorbitol-nonfermenting colonies were selected, confirmed as E. coli O157, and sent to NRL.

At NRL, both clinical and ground beef O157 isolates were confirmed as *E. coli* O157:H7, susceptible to all of the antibiotics assayed, as previously described (*9*). Genotypic characterization showed that the isolates harbored *stx*2, *eae,* and EHEC-*hly*A genes.

To establish their clonal relatedness, the strains were assayed by subtyping methods (*9*). The identity of the strains was confirmed by the unique pulsed-field gel electrophoresis (PFGE) pattern with the restriction enzymes *Xba*I and *Avr*II, and the same phage type 4. In addition, both strains were characterized as *stx*2/*stx*2vh-a by PCR-restriction fragment length polymorphism.

To our knowledge, this is the first HUS case in our country in which the source of infection was identified. No investigation was conducted to trace back the source of the ground beef. This study illustrates the importance of the surveillance of STEC infections and the usefulness of molecular subtyping techniques, such as PFGE and phage typing, to determine the relatedness of strains and assess epidemiologic associations.

The public should be made aware that hamburgers, even when prepared at home, can be a source of infection. A primary strategy for preventing infection with *E. coli* O157:H7 is reducing risk behaviors through consumer education (*10*).
